# The Influence of Parental Emotional Neglect on Assault Victims Seeking Treatment for Depressed Mood and Alcohol Misuse: A Pilot Study

**DOI:** 10.3390/jcm5100088

**Published:** 2016-10-09

**Authors:** Kylie A. Bailey, Amanda L. Baker, Patrick McElduff, David J. Kavanagh

**Affiliations:** 1 Centre for Brain and Mental Health, School of Medicine and Public Health, University of Newcastle, University Drive, Callaghan 2308, NSW, Australia; amanda.baker@newcastle.edu.au (A.L.B.); patrick.mcelduff@newcastle.edu.au (P.M.); 2 Centre for Children’s Health Research, Institute of Health & Biomedical Innovation and School of Psychology & Counselling, Queensland University of Technology, GPO Box 2434, Brisbane 4001, QLD, Australia; david.kavanagh@qut.edu.au

**Keywords:** trauma, depression, alcohol misuse, comorbidity and child neglect

## Abstract

This study explores the relationship between reported parental emotional neglect when a child, assault type experienced, posttraumatic stress symptoms (PTSS), depression, and alcohol consumption in treatment seekers for comorbid depressive symptoms and alcohol misuse. Participants (*n* = 220) with concurrent depression and alcohol misuse were recruited from the DAISI (Depression and Alcohol Integrated and Single-focussed Interventions) project. Assault type and PTSS were retrospectively assessed by the Posttraumatic Stress Diagnostic Scale. The Measure of Parenting Style is a self-report measure that retrospectively assessed emotional neglect experienced as a child. An exploratory factor analysis using the tetrachoric correlation matrix (applying principal factor extraction with a varimax rotation) identified the two assault factors of sexual assault (*SA*) and physical assault (*PA*). A path analysis revealed that Maternal Emotional Neglect increased the impact of PTSS and depression. Paternal Emotional Neglect increased the impact of *PA* on PTSS and alcohol dependence symptoms. There appears to be differential effects of assault type and Maternal/Paternal emotional neglect on depression and alcohol misuse, suggesting that parenting roles serve distinct protective functions.

## 1. Introduction

Following a traumatic event, most people will experience some posttraumatic stress symptoms (PTSS), typically intrusion (e.g., thoughts, images, or nightmares) and hyperarousal (e.g., anxiety) symptoms [1]. Other mental health symptoms such as depression [2,3] and alcohol misuse can also develop following exposure to a traumatic event [4,5]. PTSS and depression are considered to be the most commonly experienced posttraumatic psychiatric conditions [6] that interact and increase psychological distress [7]. After a traumatic event, alcohol may be used to alleviate PTSS and other distressing psychological symptoms [8]. Alcohol use in this situation may represent an avoidance strategy, in that it may help to anesthetise and delay experiencing overwhelming and distressing PTSS [9].

### 1.1. Assaults

The lifetime prevalence of experiencing sexual assault is higher for women than men [10,11,12]. People who have been sexually assaulted are also more likely to report depressive symptoms [11,13,14] and are at greater risk of alcohol use disorder [15,16,17] compared with people who have not been sexually assaulted. In particular, the prevalence rates for Posttraumatic Stress Disorder (PTSD) and depression in women who report sexual assault is 36% (respectively) while alcohol misuse is 20% [18]. Exposure to lifetime violence also often results in an increased risk of PTSD, major depressive disorder [19] and higher rates of at-risk drinking [15,20]; with victims most commonly reporting distress with the physical effects of the assault as well as heightened fear and anxiety when in public places [21].

### 1.2. Neglectful Parenting

Neglect can be defined as the unresponsive care towards a child by their caregiver, that is so chronic that it disrupts the developing brain [22]. Neglect includes inadequate nurturing and affection to the child [23] as well as constantly ignoring and denying them opportunities to mix with other people or children [24]. Research suggests that the prevalence of neglect is approximately one in ten children [25] with no gender differences in reported exposure [23].

Child neglect often co-occurs (and therefore is researched) with other forms of childhood maltreatment and adverse circumstances [26]. One large study investigated the effects of adverse circumstances in childhood on health behaviours and disease when an adult and found that a range of childhood adverse circumstance (including domestic violence, abuse and neglect) were interrelated. In particular, they found that 57.5% of participants who reported childhood emotional neglect also experienced physical abuse, whilst 36.9% also reported sexual abuse [26]. The limitation of this study was that it did not investigate later psychological effects or risks of exposure to subsequent assault, when an adult.

Other research on adults who were exposed to childhood maltreatment (including neglect) [22,27,28,29,30] report poorer physical and mental health outcomes [22] including higher levels of adult depression [31], alcohol misuse [30] and PTSD [32]. Research on children has also shown that chronic neglect in childhood is associated with more negative mental health outcomes compared with abuse exposure [33]. The negative mental health outcomes from neglect during childhood are also found in adolescents [34] and (more recently) adults [35,36,37]. The long-term effects of childhood neglect across middle and older adulthood are only now beginning to be investigated [32,38].

### 1.3. Models That Consider Impact of Childhood Emotional Neglect

Various models have been developed to explain the impact of neglect on the developing child. Such models include the Bronfenbrenner model that considers the child in the context of family, community and other ecological systems [33]. The Belsky model considers the effects of the parent and their parenting style on the child [34]. Attachment models also consider the impact of neglect through insecure and disorganised attachment styles [27]. More recently, the effects of childhood neglect has led to the development of biological stress response models [36] and brain development models [37] to explain the impact of neglect on learning, thinking and emotional regulation. Despite the growing literature on the impact of neglect on child development, research on the effects of neglect are still lacking [23]. Furthermore, there is no model that has been developed to explore the pathway of childhood emotional neglect, and future assaults, depressive symptoms and alcohol misuse in adult mental health populations.

### 1.4. Summary

Past research investigates the effects of co-occurring posttraumatic psychiatric conditions, rather than the sequencing of symptoms. For example, sexual and physical assaults are both associated with PTSD [39,40] and PTSD often co-occurs with depressive and alcohol use disorders [6,20,38]. Parental emotional neglect studies have also shown associations with adult PTSD [29], depression [31] and alcohol misuse [41] but tend to assess emotional neglect in combination with other forms of abuse/maltreatment [27,29,30,35]. Relationships and predictive pathways between parental neglect and traumatic assaults, with comorbid PTSS, depression, and alcohol misuse in adult mental health populations, are limited. It is also unknown whether exposure to parental emotional neglect can lead to vulnerabilities in experiencing particular traumatic assaults as well as particular psychological symptoms.

### 1.5. Current Study

This study aimed to expand on knowledge about the effects of parental emotional neglect on participants seeking treatment for depressed mood and alcohol misuse (who report assault exposure) by capitalising on the DAISI (Depression and Alcohol Integrated and Single focussed Interventions) project’s baseline data from Baker, et al. [42,43]. The DAISI project was a randomised controlled trial conducted in Newcastle and Brisbane, Australia, evaluating four different psychological interventions for co-existing depression and alcohol misuse. In Stage 1, we aimed to explore the relationships between parental emotional neglect as a child and exposure to sexual/physical assaults (regardless of age of exposure) on severity of PTSS, depression, drinking and alcohol misuse. In Stage 2, we used a path analysis to develop separate exploratory and predictive models for the effects of parental emotional neglect on exposure to sexual/physical assaults as well as severity of PTSS, depressive and alcohol dependence symptoms. We hypothesised that, compared with those who did not report assault exposure, participants who reported assault exposure would be more likely to report more parental neglect during childhood; and in adulthood, and would show more severe PTSS, depression and alcohol misuse.

## 2. Experimental Section

### 2.1. Participants

Participants were recruited into the DAISI project [42] following approval by both the Human Research Ethics (University of Newcastle) and the University of Queensland Ethics Committees (ethics approval code: H-066-0705). Recruitment was via a range of treatment agencies and media advertisements. Inclusion criteria were: (a) ≥16 years of age; (b) current depressive symptoms (score ≥17 on the Beck Depression Inventory-II, BDI-II); (c) and consuming alcohol at harmful levels as determined by the Australian National Health and Medical Research Council’s (2001) drinking guidelines [44]. Potential participants were excluded if they: (i) were currently diagnosed with a psychotic disorder; (ii) reported a history of traumatic brain injury; (iii) lacked fluency in English; or (iv) lived too far away to attend sessions.

Ages of participants ranged from 20 to 73 years, with an average of 45.3 (*SD* 11.0). There were 113 men and 107 women, with the majority experiencing a traumatic event (71.8%, *n* = 158) [38].

### 2.2. Measures

Traumatic exposure and PTSS severity (in the past month) were measured using the Posttraumatic Diagnostic Scale (PDS). Traumatic exposure was assessed using the PDS 12 traumatic event checklist, which has 11 specific trauma categories and one ‘other’ [45]. For an event to be considered traumatic, it must also meet the DSM-IV PTSD Criterion A of feeling helpless or hopeless during traumatic exposure [46]. PTSS severity was measured by summing items 22–38, which were ranked from ‘5 or more times a week’ (3), ‘2 to 4 times a week’ (2), ‘once a week or less’ (1), and ‘not at all or only once’ (0). The score ranges between 0 and 51. The internal consistency of the total PTSS summed score is (α) 0.92 with the higher the summed score, the higher the PTSS severity [45]. The PDS has high internal consistency (Chronbach α = 0.92), good test-retest reliability (κ = 0.74), sensitivity (82.0%), and diagnostic agreement (79.4% agreement, κ = 0.59 [45].

The Measure of Parenting Style (MOPS); assessed experiences of dysfunctional parenting during childhood, with the three separate scales of ‘Indifference’ (parental neglect, items 5, 8, 10, 11, 12, and 13), ‘Over-control’ (items 1, 3, 4 and 6) and ‘Abuse’ (items 2, 7, 9, 14 and 15) from both parents, separately [47]. Participants rated ‘how true’ the 21 items were in relation to their mother’s and father’s behaviour towards them, through to 16 years of age. Each parent’s behaviour was rated as ‘extremely true’ (3), ‘moderately true’ (2), ‘slightly true’ (1) or ‘not true at all’ (0). All scores were summed with higher scores denoting more dysfunctional parenting. The potential ranges for the subscales were 0–18 for Neglect, 0–12 for Over-control and 0–15 for Abuse [47].

Depressive symptoms for the previous 2 weeks were measured using the 21-item Beck Depression Inventory (BDI-II) [48]. BDI–II scores range from 0 to 63. The BDI-II has good reliability (α = 0.92), and is able to distinguish depressed from non-depressed populations [48].

Alcohol misuse during the previous 6 months was measured with the Alcohol Use Disorders Identification Test (AUDIT) [49]. The AUDIT has 10 items with a potential range of 0–36 with questions 1–3 assessing for levels of alcohol consumption, questions 4–6 assessing alcohol dependence symptoms, and questions 7–10 assessing for harmful alcohol use [50]. The higher the summed score, the more severe the alcohol misuse (score range = 0–36). Sensitivity and specificity of the cut-off score of 8 was more than 0.90 [50].

The Severity of Alcohol Dependence Questionnaire (SADQ-C) assessed alcohol dependence symptoms in a heavy drinking period within the previous 6 months. The score range is 0–60 [51]. It has sound reliability and validity, and a cut-off of 4 indicates mild alcohol dependence [52].

Drinking frequency was assessed by the Alcohol Timeline Followback (TLFB) method, focusing on the previous two weeks [53]. TLFB is a calendar method that accurately and retrospectively measures daily alcohol consumption (and the variability in consumption levels) [54]. It has high test-retest reliability with coefficients ranging from 0.79 to 0.96 over a 30- or 90-day period [53].

### 2.3. Procedure

Two 1-h assessment appointments a week apart were made. Participants received AUD$20 as reimbursement for travel costs.

### 2.4. Statistical Analysis

Factor analysis was performed as a data reduction exercise on the 12 traumatic events listed in the PDS. It used the tetrachoric correlation matrix (due to traumatic events being dichotomous) and applied principal factor extraction with a varimax rotation. The analysis identified the following four trauma event factors with eigenvalues >1: *Sexual Assault* (*SA:* including family sexual assault, stranger sexual assault, and sexual contact <18 years); *Physical Assault* (*PA:* gaol, and family and stranger non-sexual assault); *Combat and Torture (CT)*; and *Serious Accident and Natural Disaster (SAND)*. Preliminary analyses showed no significant differences in depression symptom severity, alcohol misuse and other psychological symptoms between the *SAND* group with the participants in the DAISI study that reported no trauma exposure (perhaps because many in this group were exposed to low-grade trauma during the 1989 Newcastle earthquake). The *CT* (*n* = 12) group numbers were too small to be included in the analysis. Accordingly, this study focused on whether participants reported *SA* or *PA* (due to the factor analysis process identifying them as separate factors) and to determine if there were symptomatic differences between the two assault groupings. For further information about this analysis, please see Bailey et al. [38].

This investigation was divided into two stages. The first assisted in identifying different symptom associations and relationships for *SA* and *PA* by using Spearman rho (ρ) correlations and ordinal logistic regressions. This cautious analysis approach was selected due to the data being mildly skewed, and the ranked nature of the traumatic event types within each assault group (0, no Assault; 1, one assault type; 2, two assault types; and 3, three traumatic assault types experienced). To control the family-wise error (when examining a large set of correlations/comparisons) we used the Benjamini and Hochberg false discovery rate approach [55]. Thus, *SA* and *PA* analysis had statistical significance set at 0.002 and 0.003 respectively, to control for multiple comparisons in correlations. The ordinal logistic regression adopted a stepwise approach due to the exploratory nature of this study so that the best model could be developed. Missing data was mostly for the MOPS assessment and were managed through the list-wise deletion of cases [56].

The second stage explored the pathways (through path analysis) between Maternal/Paternal Emotional Neglect and assault exposure (as the dichotomous variables for *SA* and *PA*) with severity of PTSS, and symptoms of depression and alcohol dependence. The models of *SA* and *PA* were developed by applying the goodness of fit for each model. All analysis was conducted using SPSS for Windows (version 19.0). AMOS Graphics [56] software was used to conduct the *SA* and *PA* path analysis.

## 3. Results

### Sample Characteristics

Demographic characteristics for *No Trauma*, *Trauma Experienced (by all participants recruited from the DAISI study)*, *SA* and *PA* groups are displayed in Table 1. Seventy-four (38.7%) participants reported exposure to *SA*, with: 33 (15.0%) reporting exposure to one *SA* event type: 28 (12.7%) reporting two *SA* event types; and 13 (5.9%) reporting all three *SA* event types. Eighty-four (44.0%) participants reported exposure to *PA*, with: 54 (24.5%) reporting exposure to one *PA* event type; 21 (9.5%) reporting two *PA* event types; and 9 (4.1%) reporting exposure to all three *PA* event types. Of the participants reporting assault exposure, 46 (24.2%) reported exposure to both *SA* and *PA*. The only significant demographic difference within each of these groupings was that there were more females than males in the *SA* group (74.3% vs. 25.7%; χ^2^ (1, 190) = 33.93, *p* < 0.001). Females also reported more severe Maternal Emotional Neglect scores, compared with males (*t*(171) = 2.8, *p* = 0.007).

#### Stage 1: Correlations and Regressions of Sexual and Physical Assault Variables.

The *SA* and *PA* Spearman’s rho (ρ) trauma analysis had statistical significance set at 0.002 and 0.003 respectively, to control for multiple comparisons in correlations. *SA* was ranked as: no *SA* (0), one *SA* type (1), two *SA* types (2), and three *SA* types (3). *PA* was ranked as: no *PA* (0), one *PA* type (1), two *PA* types (2), and three *PA* types (3).

***Sexual Assault.*** An increase in number and type of *SA* was associated with more severe PTSS (ρ = 0.25, *p* = 0.001). Compared with those who had not been sexually assaulted, *SA* was associated with Maternal Emotional Neglect (ρ = 0.32, *p <* 0.001), Maternal Over-control (ρ = 0.26, *p* = 0.001), and Maternal Abuse (ρ = 0.28, *p <* 0.001) and also with Paternal Emotional Neglect (ρ = 0.27, *p* = 0.001) and Paternal Over-control (ρ = 0.25, *p* = 0.002). *SA* was found to be significantly associated with earlier depressive episode onset (ρ = −0.23, *p* = 0.002), and having more severe depressive symptoms (ρ = 0.24, *p* = 0.001).

Ordinal logistic regression found a significant relationship effect of Maternal Emotional Neglect on *SA* (WALD χ^2^ (1) = 5.85, *p* = 0.02) and having more severe PTSS (WALD χ^2^ (1) = 6.19, *p* = 0.01) and depressive symptoms (WALD χ^2^ (1) = 4.13, *p* = 0.04). The goodness of fit statistic (ρ = 0.38) supported these findings, while the test of parallel lines showed that the proportional odds assumption had not been violated (χ^2^ (6) = 5.24, *p* = 0.51).

***Physical Assault.*** Compared with those without *PA*, participants with *PA* experiences were more likely to have experienced Maternal Emotional Neglect (ρ = 0.27, *p* = 0.001) and Maternal Abuse (ρ = 0.27, *p* = 0.001), and Paternal Emotional Neglect (ρ = 0.39, *p <* 0.001) and Paternal Abuse (ρ = 0.29, *p <* 0.001). *PA* was also significantly associated with an earlier onset of depressive episodes (ρ = −0.25, *p <* 0.001), greater weekly drinking (ρ = 0.26, *p* = 0.001), binge drinking (ρ = 0.29, *p <* 0.001), alcohol misuse (ρ = 0.22, *p* = 0.002), and more severe alcohol dependence (ρ = 0.22, *p* = 0.003).

Ordinal logistic regression showed a significant relationship effect of Paternal Emotional Neglect on *PA* (WALD χ^2^ (1) = 16.71, *p* < 0.001) and reporting higher weekly drinking (WALD χ^2^ (1) = 8.57, *p* = 0.003) and earlier onset of depression (WALD χ^2^ (1) = 5.98, *p* = 0.01). The goodness of fit statistic (ρ = 0.81) supported these findings, while the test of parallel lines showed that the proportional odds assumption had not been violated (χ^2^ (6) = 2.60, *p* = 0.86).

#### Stage 2: Path Analysis for SA and PA.

**Figure 1:**
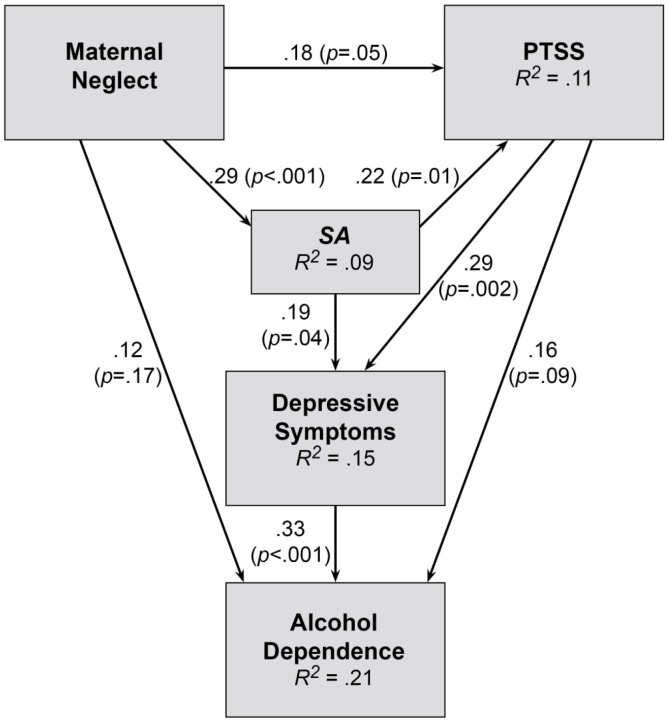
Sexual assault (*SA*) Path Analysis Model: Effects of *SA* and Maternal Neglect on symptoms of posttraumatic stress, depression and alcohol dependence.

**Figure 2:**
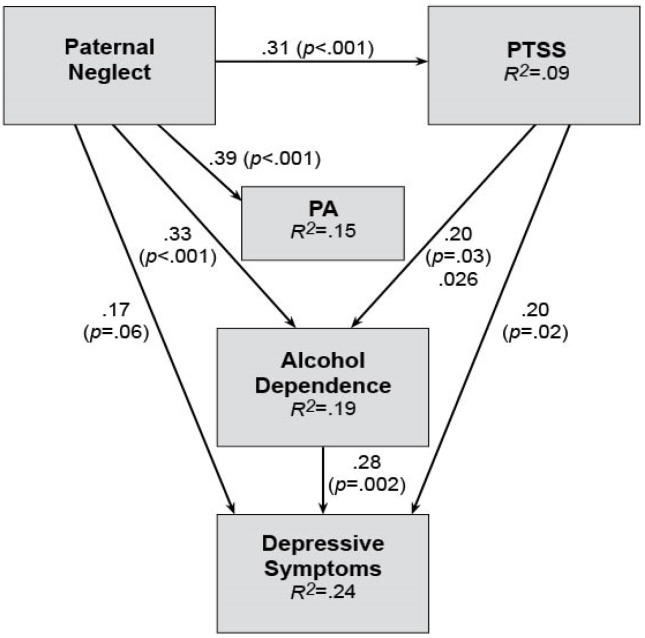
Physical assault (*PA*) Path Analysis Model: Effects of *PA* and Paternal Neglect on symptoms of posttraumatic stress, depression and alcohol dependence.

A covariance path analysis was used to test the goodness of fit of models for *SA* and *PA* separately (Figure 1 and Figure 2). The weekly drinking variable was initially included in both *SA* and *PA* models (as participants in the DAISI project were seeking treatment for coexisting depression and alcohol misuse) but was then replaced with the alcohol dependence symptom variable as it fitted both models better. This may be due to the alcohol dependence symptom variable assessing for a wide range of drinking behaviours (not simply alcohol consumption), which may make it a better indicator of the severity of alcohol misuse.

The *SA* model (χ^2^ = 1.58, *df* = 2, *p* = 0.45, GFI = 0.99, RMSEA = 0.00; Figure 1) shows that Maternal Emotional Neglect significantly increased the risk of being sexually assaulted (*p* = 0.001) and also increased the severity of PTSS (*p* = 0.05). There was a direct link to reporting more severe alcohol dependence symptoms (*p* = 0.17). Even though this direct link was not significant, it was included in the final model, as it improved its goodness of fit. *SA* exposure predicted more severe PTSS (*p* = 0.02) and more severe depressive symptoms (*p* = 0.04), whilst PTSS predicted more severe depression (*p* = 0.002) and alcohol dependence symptoms (*p* = 0.09). More severe depression was a strong predictor of more severe alcohol dependence symptoms (*p* < 0.001).

The *PA* model (χ^2^ = 0.67, *df* = 3, *p* = 0.88, CFI = 0.10, RMSEA = 0.00; Figure 2) shows that Paternal Emotional Neglect significantly increased the risk of *PA* (*p* < 0.001). Paternal Emotional Neglect also increased severity of PTSS (*p* < 0.001) and alcohol dependence symptoms (*p* < 0.001) and had a direct link to depression (*p* = 0.06). Even though the link to depressive symptoms was not significant, it was retained in the model as it improved the goodness of fit. Severity of alcohol dependence symptoms predicted more severe depression (*p* = 0.002). Severity of PTSS predicted more severe alcohol dependence (*p* = 0.03) and depressive symptoms (*p* = 0.02). The *PA* link to PTSS was removed to improve the goodness of fit. This model was also run with gender as a possible confounder, but no significant impact of gender was found.

## 4. Discussion

This exploratory pilot study is the first to investigate the long-term effects of emotional neglect by the mother and the father, on trauma sequelae for *SA* and *PA* in participants seeking treatment for concurrent depression and alcohol misuse. This is important as the long-term effects of maternal and paternal emotional neglect, combined with the effects of *SA* and *PA* on psychological symptoms is limited. In this study, we found that parental neglect as a child is associated with assault exposure and increased symptom severity; as well as identifying two separate psychological responses to assault type. In the *SA* investigations, we found that experiencing *SA* was significantly related to an earlier onset of depression, and to having more severe PTSS and depression. Compared with *SA*, *PA* exposure was significantly related to Paternal Emotional Neglect, greater weekly alcohol consumption, and an earlier onset of depression.

The *SA* path analysis model confirmed that *SA* events are related to Maternal Emotional Neglect, PTSS and depressive symptoms. From this very preliminary model, it may be proposed that when a child is neglected by their mother, they may be more vulnerable to sexual assault as the neglected child may not be protected from at-risk situations or learn protective behaviours to prevent (re-) victimisation when an adult. Maternal Emotional Neglect may also exacerbate PTSS and depressive symptoms, following *SA*. This may be due to the child not learning the psychological skills of how to self-care, self-soothe, or other skills that help to manage distress [35] as the mother was absent or indifferent. They may also not receive positive social support post-assault from their mother to help with coping, as those who report child sexual abuse also report lower levels of parental care [57] or support [58].

Our model also showed that *SA* and PTSS both contribute to depressive symptoms, which may be consistent with an ‘assault-related depression’ as depression commonly occurs when exposed to a traumatic event [1,3]. In the model, PTSS also contributes to alcohol dependence symptoms and Maternal Emotional Neglect. This result suggests that alcohol may be used to manage/self-medicate both PTSS and depressive symptoms, possibly due to inadequate life, coping, and emotional regulation skills [8,9]. This finding may also be due to the higher prevalence of depression compared to alcohol misuse, in victims of *SA*. Despite these findings of this analysis, further research is required that can both: control for recall bias regarding childhood neglect: and recruit a larger sample size to confirm this sequencing of symptom presentation for survivors of *SA*.

Stage 1 analysis on *PA* showed that *PA* exposure was associated with Paternal Emotional Neglect and Paternal Abuse, earlier onset of depression and drinking, increased weekly and binge drinking, and more severe problems with alcohol and alcohol dependence symptoms. Of concern, this analysis suggests that alcohol misuse may be the consequence of this assault type. Continued intoxication from increased alcohol use may also place the person at further risk for assault (i.e., secondary re-traumatisation) [15]. It may also increase the risk of their engagement in violent behaviours as heavy alcohol consumption is strongly associated with violence [59].

The *PA* path analysis showed that Paternal Emotional Neglect could predict *PA* and severity of PTSS and alcohol dependence. PTSS did contribute to both symptoms of alcohol dependence and depression, which is not surprising given the associations between these three disorders [60] as well as the bidirectional interaction between PTSD and alcohol [61]. The significant association between symptoms of alcohol dependence and depression may be due to the physiological effects that alcohol can have on increasing depressive symptoms [62]. It may also be due to both the study population seeking treatment for comorbid depression and alcohol misuse, as well as the bidirectional link between these two disorders [63].

What is notable in the *PA* model is that it suggests that being neglected by the father plays a role in exposure to physical assault as well as being more at risk of developing, PTSS, depression and problems with drinking. Overall, the *PA* model findings are unique and warrant further investigations to explore the protective impact of fathering on assaults, as well as comorbid mental health symptoms.

The findings of this pilot study also have public policy and clinical implications. For example, child protection policies and interventions may need to better detect and prioritise emotional neglect when working with vulnerable children. Increasing the recruitment of fathers and fathers-to-be into parenting services is a possible consideration, due to the potential protective impact they may have on future assault exposure. In regards to treatment seeking adults who report exposure to parental emotional neglect, case formulation may need to include skills training, as well as emotional literacy and regulation training to further enhance psychological treatment outcomes.

The primary limitation of this study is that the DAISI study addressed depression and alcohol misuse, resulting in some missing data for the MOPS assessments. The impact of missing data may be reduced by recruiting trauma participants only, rather than a non-trauma population that are seeking treatment for concurrent depression and alcohol misuse symptoms. Participants’ age at the time of the assault was not recorded, and other formal childhood abuse assessments were not applied, as the data relied on self-reports. Furthermore, participants who nominated both a sexual or physical assault event, were included in both the *SA* and *PA* groups (*n* = 46, 24.2%). Limitations to generalisation were that the study sample involved depression and alcohol misuse treatment seekers, and that the *SA* model was based primarily on females. The study is further limited as the data only measures one time point (i.e., cross-sectional) where by a longitudinal study may better explain associations between childhood neglect, risk of assault exposure and trauma comorbidity, overtime.

Due to these limitations, the two models are to be viewed with much caution as the symptom pathways identified may be an artifact of this particular data set (i.e., comorbid depressed and alcohol misuse client populations). The models also show associations between symptoms and experiences (neglect and assault) but do not include the bidirectional nature of PTSS, depression and symptoms of alcohol dependence. Instead, our exploratory models focus on the effects of exposure to parental neglect (by either parent, when a child) and how this neglect may be associated with assault type and the comorbid symptoms of PTSS, depression and alcohol dependence. Further research into the development of parental neglect models could draw on a larger sample size as well as consider the bidirectional nature of depression, alcohol misuse and PTSS.

This study proposes that symptom pathways exist for comorbid depressed and alcohol misuse populations who experience *SA* and *PA*, and that these pathways are significantly influenced by experiencing neglect as a child. Research is required to further explore these preliminary path analysis findings, particularly the effects of Paternal Emotional Neglect on later physical assault exposure and psychiatric morbidity. The *SA* Model also needs further developed on male *SA* victims. Research could also identify if emotional regulation and emotional literacy skills training could further benefit the treatment of psychological symptoms in adults who report parental neglect as a child. Other research could also continue to develop symptom pathway models in client populations who report both parental neglect (when a child) and assault; and consider any variations between trauma client groups and other mental health populations.

## Figures and Tables

**Table 1:** Demographic Characteristics for No Trauma, Trauma Experienced, Sexual Assault and Physical Assault Groupings (*n* = 220).

Trauma Event Type	*n* (%)	Gender	Finished School	Welfare Recipient	Marital Status (Single)	PTSD Diagnosis	Depression Onset	Alcohol Initiation
		*n* (%)	*n* (%)	*n* (%)	*n* (%)	*n* (%)	*M* (*SD*)	*M* (*SD*)
**No Trauma**	62 (28.2%)	^a^ M 33 (53.2%)	24 (39.3%)	22 (35.5%)	17 (27.4%)	--	29.7 years (14.2)	16.0 years (5.3)
		^b^ F 29 (46.8%)						
**Trauma Experienced**	158 (71.8%)	^a^ M 80 (50.6%)	78 (49.7%)	79 (50.6)	43 (27.2%)	83 (52.5%)	24.7 years (14.3)	15.1 years (5.1)
		^b^ F 78 (49.4%)						
**Sexual Assault**	74 (38.7%)	^a^ M 19 (25.7%)	35 (45.9%)	36 (49.3%)	20 (25.7%)	43 (58.1%)	21.7 years (13.2)	14.6 years (5.8)
		^b^ F 55 (74.3%)						
**Physical Assault**	84 (44.0%)	^a^ M 42 (50.0%)	43 (51.2%)	48 (57.8%	30 (35.7%)	48 (57.1%)	21.7 years (13.0)	14.5 years (5.2)
		^b^ F 42 (50.0%)						

Note. ^a^ M = male; ^b^ F = female; -- = No PTSD diagnosis for this event type.
